# Supplementary figures and images for: Transcriptome Analysis of Peripheral Blood Mononuclear Cells in SARS-CoV-2 Naïve and Recovered Individuals Vaccinated With Inactivated Vaccine
Source: Front Cell Infect Microbiol. 2022 Feb 3;11:821828. doi: 10.3389/fcimb.2021.821828 (PMC8851474; doi:10.3389/fcimb.2021.821828)

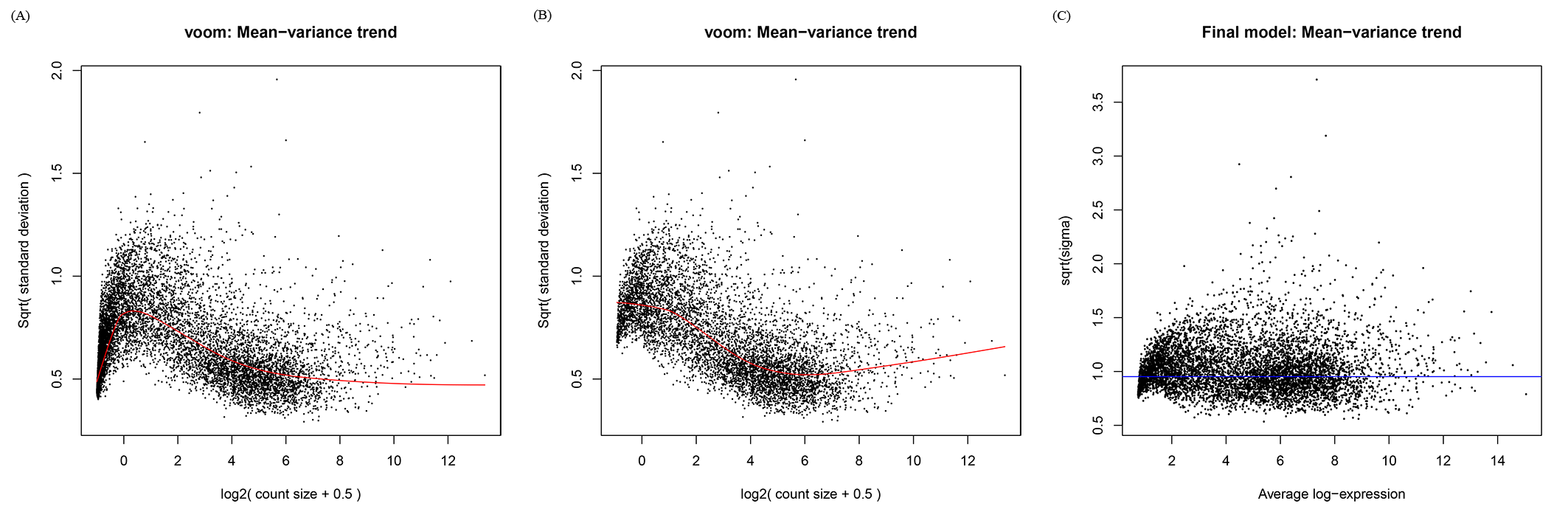

Supplement: Supplementary Figure 1 — The standardization and filtering processes of gene expression. (A) The fitting relationship between the average expression of unprocessed raw data (red line) and Sqrt. (B) The fitting relationship between the average expression of normalized and filtered data (red line) and Sqrt. (C) The linear fitting model constructed by Bayesian method. The horizontal blue line represents the average log2 residual standard deviation. [file Image_1.tif]
