# Supplementary material for: Transcriptome Analysis of Peripheral Blood Mononuclear Cells in SARS-CoV-2 Naïve and Recovered Individuals Vaccinated With Inactivated Vaccine
Source: Front Cell Infect Microbiol. 2022 Feb 3;11:821828. doi: 10.3389/fcimb.2021.821828 (PMC8851474; doi:10.3389/fcimb.2021.821828)
Supplement: Supplementary file 2 [file Table_1.doc]

**Table SI. Sequences of primers used.**

| Gene Symbol | primer-Forward (5'-3') | primer-Reverse (5'-3') |
| --- | --- | --- |
| IL1B | CCAGGGACAGGATATGGAGCA | TTCAACACGCAGGACAGGTACAG |
| CXCL8 | CACTGTGTGTAAACATGACTTCCAA | TGTGGTCCACTCTCAATCACTCTC |
| IL10 | GAGATGCCTTCAGCAGAGTGAAGA | AGTTCACATGCGCCTTGATGTC |
| JUN | CAAGAACTCGGACCTCCTCAC | CCGTTGCTGGACTGGATTATCA |
| VEGFA | GAGCCTTGCCTTGCTGCTCTA | CACCAGGGTCTCGATTGGATG |
| CXCL10 | GGCCATCAAGAATTTACTGAAAGCA | TCTGTGTGGTCCATCCTTGGAA |
| SELL | GGAAATGTCAGAGCACCCAGAG | GGTAAGTCCAGCAGTCGGTTC |
| TLR9 | CCGTGACAATTACCTGGCCTTC | CAGGGCCTTCAGCTGGTTTC |
| CCR5 | TCAGGGATAGCACTGAGCAAAG | GCCTATTGACGGTTAAATGCAGA |
| HIST1H4F | TGTCTTCGATCATGTCTGGTAGAG | GTATGTTGTCACGCAGCACTT |
| ACTB | TGGCACCCAGCACAATGAA | CTAAGTCATAGTCCGCCTAGAAGCA |
| β-actin | CCTGGCACCCAGCACAAT | GCCGATCCACACGGAGTACT |
