# Supplementary material for: Transcriptome Analysis of Peripheral Blood Mononuclear Cells in SARS-CoV-2 Naïve and Recovered Individuals Vaccinated With Inactivated Vaccine
Source: Front Cell Infect Microbiol. 2022 Feb 3;11:821828. doi: 10.3389/fcimb.2021.821828 (PMC8851474; doi:10.3389/fcimb.2021.821828)
Supplement: Supplementary file 3 [file Table_2.doc]

**Table SII. Thermocycling conditions for reverse transcription-quantitative PCR.**

| Step | Temperature (˚C) | Reaction time (sec) | Number of cycles |
| --- | --- | --- | --- |
| Initial denaturation | 95 | 30 | 1 |
| Denaturation | 95 | 10 | 40 |
| Annealing | 60 | 30 |
| Elongation | 95 | 15 | 1 |
| Final extension | 60 | 60 |
|  | 95 | 15 |
